# Preparation and Properties of Carboxymethyl Chitosan/Alginate/Tranexamic Acid Composite Films

**DOI:** 10.3390/membranes9010011

**Published:** 2019-01-08

**Authors:** Qing-Kun Zhong, Ze-Yin Wu, Ya-Qi Qin, Zhang Hu, Si-Dong Li, Zi-Ming Yang, Pu-Wang Li

**Affiliations:** 1 School of Chemistry and Environmental Science, Guangdong Ocean University, Zhanjiang 524088, China; 13924405077@163.com (Q.-K.Z.); guazi.wu@foxmail.com (Z.-Y.W.); Qmumuyi@163.com (Y.-Q.Q.); sidongligdou@163.com (S.-D.L.); 2 Agricultural Product Processing Research Institute, Chinese Academy of Tropical agricultural Sciences, Zhanjiang 524001, China; yangziming2004@163.com

**Keywords:** carboxymethyl chitosan, sodium alginate, tranexamic acid, composite films, hemostatic activity

## Abstract

In this study, the porous composite films of carboxymethyl chitosan/alginate/tranexamic acid were fabricated, with calcium chloride as the crosslinking agent and glycerin as a plasticizer. The composite films were characterized by scanning electron microscopy (SEM) and Fourier transform infrared (FTIR) spectroscopy. The properties of the composite films, including water absorption, air permeability, and cumulative release rate, were tested. In addition, their hemostatic performance was evaluated. The results showed that the appearance of the films with good adhesion was smooth and porous. FTIR showed that chemical crosslinking between carboxymethyl chitosan and sodium alginate was successful. The excellent cumulative release of tranexamic acid in the composite films (60–80%) gives the films a significant procoagulant effect. This has good prospects for the development of medical hemostasis materials.

## 1. Introduction

The skin is the first barrier of the human body for defense against external stimuli and damage. Damage caused by mechanical factors can lead to microbial invasion, to loss of protein, water, and blood volume, and even to death [1,2]. Therefore, the development of excellent films for skin trauma and rapid hemostasis is key to treatment, which is closely related to human health [3].

Chitosan (CS) is a product obtained by the deacetylation of chitin, which may be extracted from shrimp and crab shells [4]. Owing to its excellent biocompatibility, biodegradability, non-toxicity, and various physiological functions such as hemostasis, bacteriostatic, anti-cancer, and lipid-lowering [5,6], chitosan has been extensively used in biomedicine. Carboxymethyl chitosan (CMCS) is a kind of secondary derivative obtained by the carboxymethylation of chitosan [7]. Due to the coexistence of -NH_2_ and -COOH, CMCS has better biocompatibility, moisture absorption, antibacterial, and film-forming properties compared to chitosan [8,9]. Sodium alginate (SA) is a natural polysaccharide extracted from kelp or seaweed. As a natural wound repairing material, sodium alginate has good thickening, flocculation, and chelating properties [10]. Due to its unique film-forming properties and non-toxic degradability, it is widely used as a gel film and clinical medical dressing which has healing effects on burns, piercing wounds, and deep ulcer bleeding [11,12]. Tranexamic acid (TA) is a synthetic antifibrinolytic drug which has been used clinically in the treatment of bleeding caused by fibrinolysis [13,14,15]. Its structure is similar to that of lysine, which inhibits the cleavage of fibrin clots by competitively inhibiting the binding of fibrin lysine to plasmin, thereby producing hemostasis. However, TA has a short half-life and low bioavailability. It is an effective approach to controlled drug release by using polymer as a carrier. To the best of our knowledge, the combination of tranexamic acid and polymer matrix as a new hemostatic material has been seldom studied.

In this paper, sodium alginate and carboxymethyl chitosan were used as the carrier matrix and a hemostatic active ingredient tranexamic acid was added to prepare the composite films which had good sustained release effects. The composite films are expected to be used for clinical hemostasis.

## 2. Materials and Methods

### 2.1. Materials

Carboxymethyl chitosan, sodium alginate, tranexamic acid, glycerin, and anhydrous calcium chloride were all purchased from Sinopharm Chemical Reagent Co., Ltd. (Shanghai, China). Unless otherwise specified, all reagents were of analytical grade.

### 2.2. Fabrication of the Composite Films

Solutions with a total volume of 6 mL of SA (2%, *w*/*v*) and CMCS (2%, *w*/*v*) were mixed at volume ratios of 1:1, 1:2, 2:1, 1:5, 5:1, pure CMCS, and pure SA, and were recorded as samples 1#, 2#, 3#, 4#, 5#, 6#, and 7#, respectively. After being stirred fully, 0.5% CaCl_2_ solution (2 mL) was added to crosslink and the mixture was frozen overnight. The mixture was washed repeatedly with absolute ethanol three times and the matrix films were formed. The matrix films were then put into a solution of tranexamic acid (0.01 g/mL)–10% glycerol and soaked for 30 min. The composite films were obtained by lyophilization at −60 °C for 24 h.

### 2.3. Appearance Observation

The appearance of the composite films was recorded with a digital camera and a scanning electron microscope (SEM) (Hitachi S-4800, Tokyo, Japan). The pretreatment of samples for observation under the SEM was the process of gold spray. The conditions of determination were a working voltage of 1 kV and a distance of 10.3 mm.

### 2.4. Measurement of Fourier Transform Infrared (FTIR)

A Spectrum 100 Fourier transform infrared spectrometer (PerkinElmer, Waltham, MA, USA) was used to measure the samples. The films were directly used for an attenuated total reflection (ATR) accessory. The infrared spectrum was recorded over wave numbers ranging from 4000 cm^−1^ to 450 cm^−1^.

### 2.5. Determination of Water Absorption Rate

The composite films were cut into rectangular pieces and weighed. The rectangular films were placed into a beaker containing distilled water for 3 h at room temperature and then taken out. The water on the surface of the films was removed with filter paper and the films were weighed again. Tests were performed in triplicate and the results were expressed as mean ± standard deviation. The formula for the water absorption rate was

(1) $$\mathrm{Water}\;\mathrm{absorption}\;\mathrm{rate}\;(\%)\;=\frac{m_{1}-m_{0}}{m_{1}}\times 100\%$$

where, *m*_0_ and *m*_1_ were the mass of films before and after soaking, respectively.

### 2.6. Determination of Air Permeability

Two same-size Erlenmeyer flasks, each containing 100 mL distilled water, were weighed (*m*_1_). One was sealed with the composite films and the other left untreated and set as the blank control. They were placed overnight in an environment with a humidity of 43% (saturated Na_2_CO_3_ solution) at room temperature. The films were then removed and the flask which had contained them was weighed again (*m*_2_). The blank control flask was weighed as *m*_3_. A fresh-keeping film sold commercially was used as a positive control. The formula for the air permeability was

Air permeability (%) = (*m*_1_ − *m*_2_)/(*m*_1_ − *m*_3_) × 100% (2)

where *m*_1_ − *m*_2_ and *m*_1_ − *m*_3_ were the losses of water in the experimental group and in the control group, respectively.

### 2.7. Determination of Cumulative Release Percent

The composite films (50 mg) were placed in an Erlenmeyer flask and the volume was set to 50 mL using distilled water. An amount of the solution (1 mL) was removed from the flask and the same volume of distilled water was replenished at designated times (5 min, 10 min, 30 min, 1 h, 3 h and 24 h). The absorbance of the solution was measured using spectrophotometry (UV-1800, Jinghua Technology, Shanghai, China) at 220 nm and the content of tranexamic acid was calculated according to the measured standard curve formula (*C* = 54.35A + 1.76, *R*^2^ = 0.99503). The cumulative release percent (*CRP*%) was calculated as

(3) $$CRP\%=\frac{C_{j}\times V_{total}+\sum_{i=0}^{j-1}C_{i}V_{taken}}{m}\times 100\%$$

where, *C_i_* and *C_j_* were the concentrations of tranexamic acid at the moments *i* and *j*, respectively, in µg/mL; *V_taken_* and *V_total_* were the volumes of the taken and total solutions, respectively, in mL; and *m* was the total mass of tranexamic acid in the composite films.

### 2.8. In Vitro Clotting Time

The composite films (0.20 g) were placed in a test tube and kept at 37 °C. Fresh anticoagulated rabbit blood (1 mL/tube) was added and timing subsequently started. The tube was tilted every 15 s and timing continued until the blood clotted. A blank control and a positive control (Yunnan Baiyao, 1 g/mL) were set. The study was performed in accordance to the National Research Council Guide for the Care and Use of Laboratory Animals, and the protocol was approved by the Ethics Committee of Guangdong Medical University (Certificate No. SYXK20150147).

## 3. Results and Discussion

### 3.1. Morphological Observation

**Figure 1:**
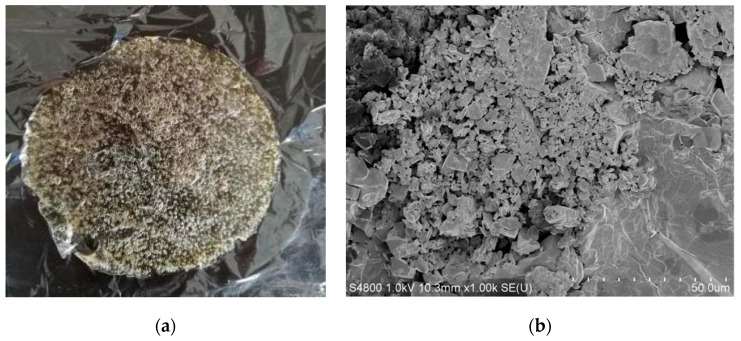
Appearances of the composite porous film 1# of carboxymethyl chitosan/alginate/tranexamic acid: (**a**) optical view, showing a porous structure and (**b**) scanning electron microscopy (SEM) micrographs, showing the loaded tranexamic acid.

The composite films of carboxymethyl chitosan/alginate/tranexamic acid were fabricated successfully. The film 1# was pale yellow and had a porous structure which was favorable for adsorbing liquid (Figure 1a). It can be seen from Figure 1b that tranexamic acid was loaded into the composite films through crystallization to increase the hemostatic property of the composite films. The appearances of other composite films (2–5#) were similar.

### 3.2. Water Absorption

High water absorbency is one of the important indicators for biological films. The water absorption results of the films are shown in Table 1. It can be seen that the pure CMCS film (6#) had the worst water absorption, while the pure SA film (7#) had the best. All the composite films (1–5#) had good water absorbency, especially sample 4#. Hydrophilicity is an important characteristic property of hemostatic biomaterials [16]. Though the content of tranexamic acid is the same, the ratio of carboxymethyl chitosan to sodium alginate is different. Water absorption is affected by the cross-linking density which is related to the amount of carboxyl groups [17]. It may be that sodium alginate has more carboxyl groups and better hydrophilicity, while the porous structure of the cross-linked composite films is more compact, which makes the water absorption slightly weaker [11].

### 3.3. Air Permeability

**Figure 2:**
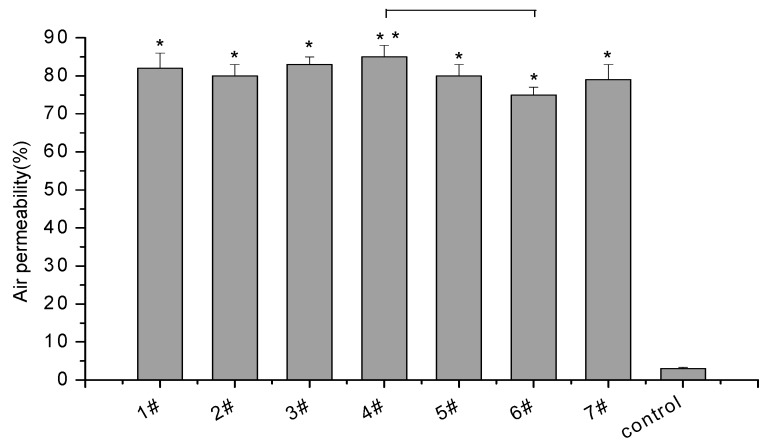
Air permeability of the composite films.

Air permeability plays an important role in medical wound healing films, and high air permeability is beneficial for wound healing. Cross-linking density has an effect on air permeability. As the cross-linking degree increases, the pore structure becomes dense and air convection is reduced. As a result, the permeability decreases [18]. Without doubt, changes in air permeability are mainly affected by the porosity of the material. The higher the porosity is, the better the gas permeability [19]. The results of air permeability are shown in Figure 2. The fresh-keeping film (Lead Mens^®^, Zhongshan Limeng Aluminum-Plastic Composite Packaging Co., Ltd., Zhongshan, China) sold commercially (the control) had poor permeability, while all the composite films (1–5#) had excellent air permeability ranging from 80% to 86%, indicating that the prepared composite films met the high permeability requirements of medical wound dressings.

### 3.4. FTIR Analysis

**Figure 3:**
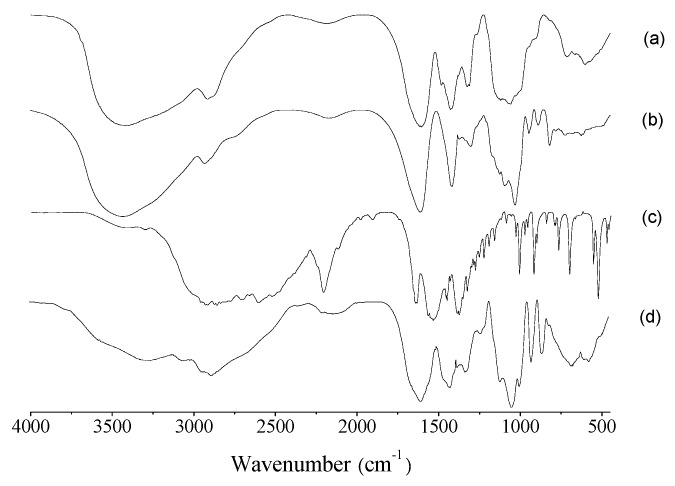
Fourier transform infrared (FTIR) spectra of (**a**) carboxymethyl chitosan (CMCS), (**b**) sodium alginate (SA), (**c**) tranexamic acid (TA), and (**d**) film 4#.

With regard to the FTIR spectrum of CMCS (Figure 3a), it can be concluded that the peak at 3417 cm^−1^ corresponded to stretching vibrations of the hydroxyl and amino groups, and that the peak at 2963–2847 cm^−1^ belonged to a –CH stretching vibration. The absorption peaks of the asymmetric and symmetrical stretching vibrations of the carboxyl group (–COOH) appeared at 1604 cm^−1^ and 1426 cm^−1^, respectively. The cluster band at 1150–1000 cm^−1^ may be said to belong to the various combinations of C-O-C stretching vibrations in the sugar rings (also shown in Figure 3c,d). As regards the spectrum of SA (Figure 3b), it can be discerned that the –OH stretching vibration is at 3422 cm^−1^, the –CH stretching vibration is at 2969–2851 cm^−1^, and the asymmetric and symmetrical stretching vibration peaks of the carboxyl group are at 1613 cm^−1^ and 1417 cm^−1^, respectively. In the spectrum of TA (Figure 3c) a strong absorption band at 1628 cm^−1^ appeared, which is the stretching vibration of C=O. The peaks at 1523 cm^−1^ can be assigned to the bending vibrations of the amino groups. The absorption peaks at 1366 and 1008 cm^−1^ are due to the asymmetrical and symmetrical stretching, respectively, of the C–C–O system. There are sharp absorption peaks at 900~650 cm^−1^ which are mainly caused by trans isomerism vibration. In the spectrum of composite film 4# (Figure 3d), except for the characteristic absorption peaks of SA and CMCS, the characteristic absorption peaks of the carboxyl group at 1613 cm^−1^ and 1417 cm^−1^ have weakened, which verifies that Ca^2+^ has replaced Na^+^ and crosslinked with the carboxyl group.

### 3.5. Cumulative Release Properties

**Figure 4:**
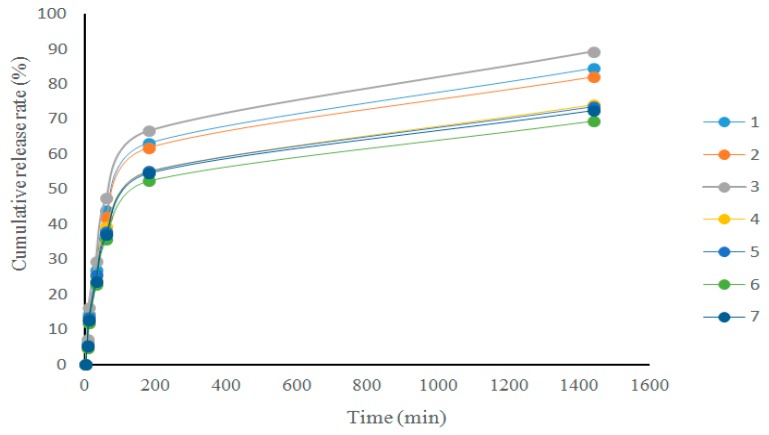
The cumulative release rate of the composite films.

The cumulative release properties of the composite films were investigated and the results are presented in Figure 4. It can be seen that the cumulative release of tranexamic acid in all the films underwent a similar trend and tended to involve a sudden release within one hour because the drug adsorbed on the surface of the films was first resolved and released. Subsequently, it involved a gentle release tendency after 200 min which was probably due to the drug inside the films being slowly released by diffusion. Film 3# had the highest cumulative release while film 6# had the lowest. Crosslinking agents played a key role in the release process. There are many crosslinking agents commonly used in polymer crosslinking such as glyoxal, glutaraldehyde, epichlorohydrin, ionic crosslinking agent, and so on. In the process of preparing the films, a crosslinking agent can be added to the film-forming solution or used in the post-treatment process of the films, which can make the structure of the films more compact and improve the mechanical and barrier properties of the films [20]. However, these chemical crosslinking agents are toxic to some extent, which may cause safety problems and other side effects. In recent years, ionic crosslinking has been used in place of chemical crosslinking [21]. Through ion crosslinking, the performance of films can be significantly improved. This may be because the structure formed by carboxymethyl chitosan and sodium alginate in proper proportions through Ca^2+^ ionic crosslinking is conducive to the release of tranexamic acid. The cumulative release rate of all the composite films was ~60–80%.

### 3.6. Procoagulant Properties

**Figure 5:**
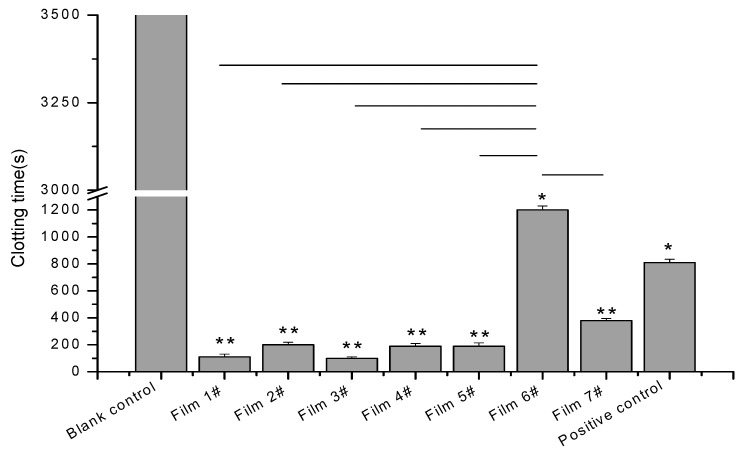
Clotting times of the composite films.

The procoagulant properties of the composite films in vitro were evaluated by the tube method. The results are shown in Figure 5 and it can be seen that the blood in the blank control group was not coagulated. It appears that film 6# (pure CMCS) gave similar results to the positive control and film 7# (pure SA) produced faster clotting, while the mixed films produced even faster clotting times (<200 s) irrespective of the ratio of SA and CMCS. In particular, film 3# promoted complete clotting of the rabbit blood within 115 s and exhibited the best coagulation effect. Films 1#, 2#, 4#, and 5# also showed excellent procoagulant effects which were superior to that of the positive control. By comparing the results of cumulative release and hemostasis, it can be found that the cumulative release rate of 3# was high and the time required for hemostasis was short. This may be due to the successful release of tranexamic acid in the composite films. The released tranexamic acid inhibits the lysis of fibrin and accelerated blood coagulation. It may also contact the blood to produce local polymerization, which promotes blood coagulation at the wound, thereby effectively stopping bleeding [22].

## 4. Conclusions

In this study, carboxymethyl chitosan/sodium alginate/tranexamic acid composite films were successfully prepared by a freeze-drying method. The composite films were porous and had good physical properties which were good for wound healing. The excellent cumulative release properties of the composite films may enhance their hemostatic effects. In particular, film 3# had the best properties and shows considerable promise for use in the development of hemostatic materials.

## Figures and Tables

**Table 1:** Water absorption rates of the composite porous films.

Film	*m*_0_ (g)	*m*_1_ (g)	Water Absorption Rate (%)
1	0.013	0.165	1169 ± 21
2	0.015	0.216	1340 ± 17
3	0.015	0.202	1246 ± 19
4	0.015	0.290	1833 ± 24
5	0.015	0.170	1033 ± 15
6	0.019	0.172	805 ± 16
7	0.016	0.325	1931 ± 20
